# Effect of Intravenous Sedation on Dental Implant Therapy Outcomes: A Single-Center Retrospective Cohort Study With Three-Year Follow-Up

**DOI:** 10.7759/cureus.104785

**Published:** 2026-03-06

**Authors:** Yasuhide Kaneko, Takahiro Murakami, Atsushi Okada, Satoshi Kai, Yoshiyuki Amari, Junichi Tsugawa, Jyoji Tanaka, Hidenori Yamaguchi

**Affiliations:** 1 Dentistry, Clinical Implant Society of Japan, Tochigi, JPN; 2 Dentistry, Clinical Implant Society of Japan, Toshima-ku, JPN; 3 Dentistry, Clinical Implant Society of Japan, Hyogo, JPN; 4 Dentistry, Clinical Implant Society of Japan, Tokyo, JPN; 5 Dentistry, Clinical Implant Society of Japan, Chiba, JPN; 6 Dental Anesthesiology, Nihon University School of Dentistry, Matsudo, JPN

**Keywords:** anxiety, dental implants, endosseous dental implantation, follow-up studies, intravenous anesthetics, treatment outcome

## Abstract

Background: Intravenous sedation (IVS) reduces anxiety during dental implant placement, but its association with mid-term peri-implant outcomes and post-treatment maintenance engagement in routine adult patients remains unclear. This study evaluated whether IVS is associated with maintenance attendance and 3-year implant therapy outcomes.

Methods: In this single-center retrospective cohort study using electronic dental records from a dental clinic in Japan, we included medically healthy non-smoking adults (≥18 years) who underwent posterior implant placement in native bone without augmentation and had ≥3 years of follow-up after definitive superstructure delivery (July 1, 2000, to May 31, 2023). Exposure was intravenous sedation with midazolam and propofol administered by an anesthesiologist versus local anesthesia alone. The primary outcome was maintenance attendance within six months after superstructure delivery (yes/no). Secondary outcomes included three-year implant survival; peri-implant clinical parameters (plaque control record [PCR] and bleeding on probing [BOP]); radiographic buccolingual bone width and horizontal bone loss; peri-implant mucositis; additional implant placement; and patient-reported outcomes. Group comparisons used Mann-Whitney U and Fisher’s exact tests (two-sided α = 0.05).

Results: Maintenance attendance was higher with IVS (96.8% vs. 63.0%). Three-year implant survival was 100% in both groups. BOP decreased from baseline in the IVS group; however, the between-group difference at three years did not reach statistical significance (p = 0.08). Horizontal bone loss was smaller with IVS (0.04 vs. 0.14 mm), and crestal bone width was greater with similar apical width. PCR improved in both groups. Additional implant placement was more frequent in the IVS group. Patient-reported outcomes favored IVS for relaxation, pain perception, and amnesia.

Conclusions: In this retrospective cohort, IVS use was associated with higher short-term maintenance attendance and more favorable patient-reported perioperative experience. Because of baseline group differences and the observational design, clinical and radiographic findings should be interpreted as associative and hypothesis-generating. Prospective multicenter studies with longer follow-up are warranted.

## Introduction

Dental implants are widely used to restore esthetics and function and offer broader indications than do fixed or removable prostheses [1-3]. Unlike conventional restorative care, limited to enamel/dentin, implant placement is surgical and often provokes anxiety caused by drilling noise/vibration, bleeding, and anticipated postoperative symptoms. In a prior case series, moderate‑to‑severe preoperative anxiety reduced patient satisfaction in 72.2% cases [4]. Psychosedation with general anesthesia or intravenous sedation (IVS) addresses this issue. Dental rehabilitation under general anesthesia or IVS can improve the quality of life and reduce dental anxiety in children [5]. General anesthesia, while appropriate for more invasive or prolonged procedures and for patients with disabilities, with reported implant survival of 94.9% in special‑needs cohorts [6], carries recognized risks and is not preferred for less invasive interventions. IVS preserves consciousness and protective reflexes and has a lower complication profile [7-9]; compared with nitrous oxide inhalation, it provides more reliable sedation and partial amnesia [10,11]. IVS has also been linked to better prosthodontic outcomes in patients with severe intellectual disabilities [12] and to improved intraoperative hemodynamic stability and stress reduction in anxious implant candidates [13,14]. However, evidence regarding mid-term peri-implant tissue outcomes and real-world maintenance engagement in routine adult implant patients remains limited.

Therefore, we conducted a retrospective cohort study comparing implant therapy performed with IVS versus local anesthesia alone. The primary objective was to compare maintenance attendance within six months after superstructure attachment between groups. Secondary objectives were to compare 3-year implant survival, peri-implant clinical parameters (PCR and BOP), radiographic buccolingual bone width and horizontal bone loss, peri-implant mucositis, additional implant placement, and patient-reported outcomes. We hypothesized that IVS would be associated with higher maintenance attendance and more favorable peri-implant soft-tissue parameters at three years.

A version of this manuscript was previously posted to the Research Square preprint server on May 8, 2024.

## Materials and methods

Study setting and design

This retrospective cohort study, reported in accordance with the STROBE statement, utilized information from the electronic dental records of a single dental clinic in Japan. We consistently applied standardized surgical and prosthodontic protocols and uniformly used one implant system (Astra Tech Implant System; Dentsply Sirona, North Carolina, USA). All IVS procedures were delivered by a single anesthesiologist using a consistent midazolam-propofol protocol with continuous monitoring. Radiographic and clinical measurements followed unified methods, and a structured 3-6-month maintenance recall program with digital records enabled reliable ≥3-year follow-up. These characteristics minimized procedural heterogeneity and information bias while reflecting routine care. We prespecified a minimum 3-year follow-up period to capture mid-term peri-implant tissue stability, peri-implant clinical parameters, and real-world maintenance engagement within a feasible timeframe for complete routine care follow-up. Accordingly, all results are framed as 3-year (mid-term) outcomes.

Data source and case identification

We queried the clinic’s electronic dental records and radiographic archives to identify all patients who received implants during the study period. We then screened records against the prespecified eligibility criteria and included all eligible cases with complete documentation through ≥3 years after superstructure attachment.

Patients

We included adult patients (≥18 years) who underwent posterior endosseous implant placement between July 1, 2000, and May 31, 2023, and had ≥3 years of follow-up after definitive superstructure attachment. Additional inclusion criteria were fixed implant-supported prostheses (single crowns or fixed bridges), absence of systemic diseases at preoperative assessment, no bone augmentation required at the index site, and non-smoking status. To minimize anatomical variability, only implants placed in native bone with adequate alveolar width and height (radiographically confirmed before surgery) were included.

Exposure (Intravenous Sedation vs Local Anesthesia Alone)

Exposure status was determined from the anesthesia record at the index implant surgery. Patients who received intravenous sedation constituted the IVS group, and those treated under local anesthesia without intravenous sedatives constituted the control group.

Sample size determination

The sample size was determined based on previous reports [15,16] using the G*Power software version 3.1.9.7 (University of Düsseldorf, Düsseldorf, Germany). With an effect size of 0.8, a significance level (α) of 0.05, and a power (1-β) of 0.80, the minimum required sample size was established at 27 patients per group. Given the retrospective nature of the study and the limited prior data to define effect sizes for all endpoints, this calculation was used as a feasibility check; secondary outcomes were interpreted as exploratory. By applying the inclusion/exclusion criteria described in the Patients section, we included all eligible cases from the clinic’s electronic records. Exposure status (IVS vs. control) was determined from the anesthesia record at the index implant surgery; there was no randomization or matching. All eligible patients were included, and the realized cohort exceeded the a priori minimum sample size.

Preoperative examination

The health status of patients was ascertained using a preoperative examination within 30 days prior to implant placement to confirm the absence of any abnormal findings suggestive of systemic disease. In addition, plaque accumulation was assessed using the O’Leary plaque control record (PCR) [17], and bleeding on probing (BOP, %) was recorded as the percentage of sites showing bleeding on gentle probing [18]. The PCR and BOP indices used in this study are publicly available (open-access), non-proprietary clinical measures described in the original publications; no license or permission was required for their use, and we did not reproduce any copyrighted scoring forms. Buccolingual alveolar bone widths (in mm) at the crestal and apical levels were measured to characterize baseline ridge anatomy and confirm adequate native bone volume before implant placement.

IVS

IVS was offered during the preoperative consultation when one or more prespecified indications were present: (1) patient‐reported moderate-to-severe dental anxiety or prior adverse experience under local anesthesia, (2) strong gag reflex, (3) anticipated long or invasive procedures, (4) difficulty remaining still or a history of vasovagal episodes, and/or (5) clinician judgment after shared decision-making. Eligible candidates were medically healthy (ASA I-II) per our inclusion criteria. Patients without these indications or who preferred local anesthesia alone were treated without IVS (control group). No premedication was administered.

In the operating room, a non-invasive sphygmomanometer, electrocardiograph, and pulse oximeter were attached to each patient to record baseline blood pressure, heart rate, and oxygen saturation, followed by the establishment of a peripheral venous line. Sedation was administered and titrated by the attending anesthesiologist using the Ramsay Sedation Scale [19], with the goal being moderate (conscious) sedation (target Ramsay 2-3). The Ramsay Sedation Scale is a publicly available (open-access), non-proprietary sedation scale; no license or permission was required for its use, and no copyrighted materials were reproduced in the manuscript. Ramsay scoring was used for intraoperative clinical management and was not analyzed as a study outcome. Because this assessment occurred during the index surgery, the anesthesiologist was necessarily unaware of subsequent postoperative implant outcomes. Oxygen was administered via a nasal cannula (standard disposable device; manufacturer and model varied over the study period and were not systematically recorded), and IV midazolam was administered, followed by a brief titration of propofol to achieve conscious sedation immediately before surgery. IVS was discontinued once the procedure started; no maintenance infusion or additional sedatives were administered intraoperatively. Following local infiltration anesthesia with 2% lidocaine hydrochloride with 1:80,000 adrenaline, implant placement was initiated. The total local anesthetic volume was typically 1.8-3.6 mL (i.e., one to two 1.8‑mL cartridges), depending on the surgical site and extent. If patients reported pain or discomfort, supplemental infiltration with the same anesthetic solution was administered as needed, together with analgesics when appropriate. Prior to the conclusion of surgery, an intravenous nonsteroidal anti-inflammatory analgesic (NSAID) was administered as a single dose for postoperative analgesia, and the implant placement procedure was completed. Postoperatively, flumazenil was administered, and patients were discharged after regaining clear consciousness.

During the procedure, electrocardiography and pulse oximetry were monitored continuously, and non‑invasive blood pressure was recorded at ≥5‑min intervals. Predefined instability criteria were SpO₂ < 94% sustained for ≥10 s despite supplemental oxygen, systolic blood pressure < 90 mmHg or > 180 mmHg or a ≥20% change from baseline, heart rate <50 or > 120 beats/min, any new clinically significant arrhythmia, or unintended deep sedation (Ramsay ≥ 4). If any criterion was met, the protocol specified immediate airway maneuvers and increased oxygen flow, verbal/tactile stimulation, and treatment of pain with additional local anesthesia; if needed, intravenous fluids and vasoactive or anticholinergic agents would be administered under an anesthesiologist's oversight, with escalation to assisted ventilation or transfer per institutional policy. Because no maintenance sedative infusion was used intraoperatively, management focused on supportive measures rather than cessation of an infusion.

Implant placement surgery and attachment of superstructure

The implant placement procedure was standardized across both groups. Patients were treated in the operating room and positioned in a dental chair (supine or semi‑supine). Routine intraoral aseptic preparation and sterile draping were performed. Implant placement was performed under local infiltration anesthesia with 2% lidocaine hydrochloride with 1:80,000 adrenaline; the total local anesthetic volume was typically 1.8-3.6 mL (one to two 1.8‑mL cartridges), depending on the surgical site and extent, with supplemental infiltration using the same anesthetic solution as needed. A mid‑crestal incision was made along the alveolar ridge, and a mucoperiosteal flap was elevated. Osteotomy preparation followed the manufacturer‑recommended drilling sequence for the implant system (Astra Tech Implant System; Dentsply Sirona, NC, USA), using a surgical guide plate under copious sterile saline irrigation. After implant placement, the wound was closed with non‑absorbable nylon sutures. Operative time and specific device models (e.g., surgical motor/handpiece) varied over the long study period and were not consistently documented in the medical records; therefore, they were not analyzed.

After 3 months of off-loading, both occlusal contact and cleaning status were confirmed using provisional restorations. The superstructure was fabricated using an open-tray method. After attaching the superstructure, intraoral radiographs were obtained using the paralleling technique to record the condition of the peri-implant bone.

Baseline and procedural characteristics

Baseline and procedural characteristics, including age at implant placement, sex, implant diameter and length categories, type of superstructure, and fixation method, were extracted from the electronic dental records. Preoperative anxiety was retrospectively assessed using the postoperative questionnaire as a baseline descriptor rather than a study outcome.

Outcomes

The prespecified primary outcome was maintenance attendance within 6 months after superstructure attachment (yes/no). Maintenance attendance was defined as attendance of at least one maintenance visit within six months after superstructure attachment, based on the clinic’s electronic records. Secondary outcomes included 3-year implant survival, peri-implant clinical parameters (PCR and BOP), radiographic buccolingual bone width and horizontal bone loss, peri-implant mucositis, additional implant placement, and patient-reported outcomes (PROs). BOP was measured at six sites per implant using a standardized probing force of 0.25 N [20]. Radiographic measurements for horizontal bone loss used periapical radiographs acquired with the paralleling technique immediately after superstructure attachment (baseline) and at 3-years, analyzed according to Kullman’s method [21]. Peri-implant mucositis was defined as BOP in the absence of radiographic bone loss [22]. PROs were recorded using a one-time, author-developed 14-item questionnaire (Appendix 1) administered at the routine 3-year follow-up visit and scored on a 5-point Likert scale (0-4). Because this questionnaire has not undergone formal psychometric validation and includes retrospective assessment of perioperative experience (including preoperative anxiety), PRO results were interpreted as exploratory. All study outcomes were defined a priori as endpoints assessed at or after the superstructure phase. The questionnaire is reproduced in full in Appendix 1; therefore, no third-party permission was required.

Statistical analysis

We assessed the distribution of continuous and ordinal variables using the Shapiro-Wilk test and visual inspection of histograms and Q-Q plots. Because several variables were non-normally distributed and patient-reported items were represented by ordinal Likert scores, non-parametric tests were used. The primary analysis compared the primary outcome (maintenance attendance within 6 months) between groups using Fisher’s exact test. Because multiple secondary endpoints were evaluated, p-values for secondary outcomes were interpreted as exploratory and no formal adjustment for multiple comparisons was applied. To ensure complete reporting, the corresponding test statistics are reported alongside p-values (Mann-Whitney U with standardized Z for continuous/ordinal variables; odds ratios with 95% confidence intervals for categorical variables analyzed using Fisher’s exact test).

For between-group comparisons (IVS vs. control), the Mann-Whitney U test was applied to the following: age at implant placement, preoperative PCR (%) and BOP (%), preoperative buccolingual alveolar bone width (mm) at crestal and apical levels, 3-year PCR (%), 3-year BOP (%) measured at six sites/implant with 0.25 N probing, 3-year buccolingual widths (crestal and apical), 3-year horizontal bone loss (mm) per Kullman’s method, and PRO item scores (Questions Q1-Q5 and Q9-Q11).

For within-group pre- vs. 3-year comparisons, the Wilcoxon signed-rank test was used for PCR and BOP in each group.

For categorical variables, Fisher’s exact test was used for sex, implant diameter category (narrow ≤ 3.5 mm vs. regular ≥ 3.6 mm), implant length category (short ≤ 8.0 mm vs. medium ≥ 8.1 mm), type of superstructure (single vs. bridge), fixation method (cement- vs. screw-retained), maintenance attendance within 6 months, peri-implant mucositis at 3 years, and additional implant placement among patients with two or more missing teeth.

All tests were two-sided with a significance level of 0.05. Analyses were performed using IBM SPSS Statistics (version 20.0; IBM Corporation, Armonk, NY, USA).

Ethical review

The Ethical Review Board of the Japanese Society of Oral Implantology approved the study (approval No. 2023-14, Medical Ethics Review Board No. 11000694) and waived the requirement for written informed consent owing to retrospective collection of de-identified data. Instead, an opt-out process was implemented. The study was conducted in accordance with the principles of the Declaration of Helsinki.

## Results

Participant selection

**Figure 1:**
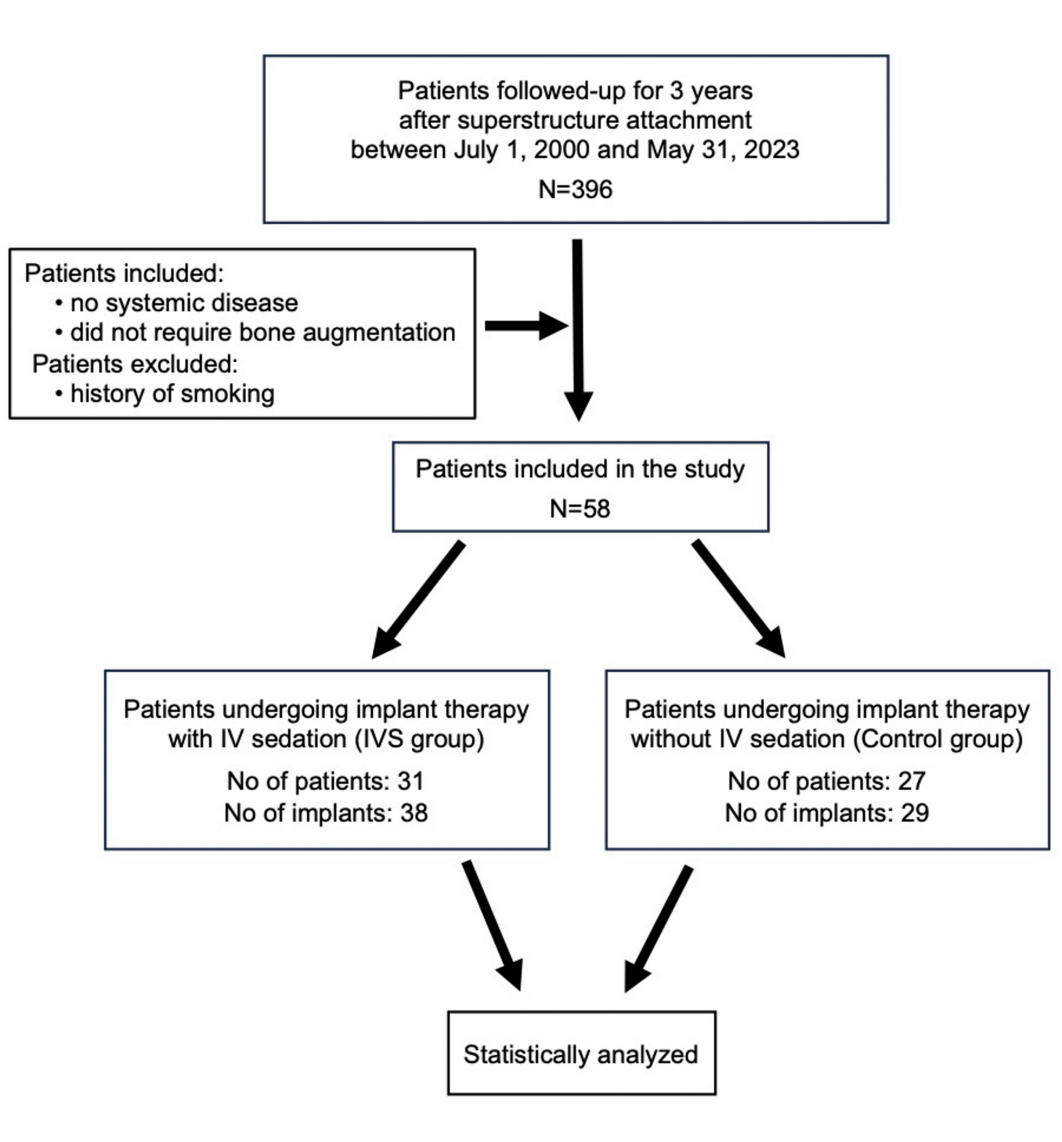
Flow chart of medical records reviewed

We screened 396 patients who had at least 3-years of follow-up after superstructure attachment between July 1, 2000, and May 31, 2023. Of these, 58 patients (67 implants) met the eligibility criteria and were included (mean age 53.83 ± 10.96 years; 41 women and 17 men). The IVS group comprised 38 implants in 31 patients, and the control group comprised 29 implants in 27 patients. The remaining patients were excluded because they did not meet one or more inclusion criteria (presence of systemic disease, requirement for bone augmentation, treatment outside the posterior region, or history of smoking). Because the screened population (n=396) consisted of patients with ≥3 years of follow-up after superstructure attachment, the high exclusion rate primarily reflected prespecified eligibility criteria rather than loss to follow-up; reasons for exclusion are summarized in Figure 1.

Participant characteristics

Patient characteristics are presented in Table 1. Notably, all bridges consisted of three-unit superstructures supported by two implants. The sample predominantly involved single-tooth restorations in posterior sites, which is relevant for interpreting mid-term outcomes.

**Table 1:** Baseline and procedural characteristics Mann–Whitney U test was used for analysis. *P < 0.05. Data are expressed as mean (± standard deviation). A statistically significant difference was found between *, **, and ***. BOP: Bleeding on probing; IVS: Intravenous sedation; PCR: Plaque-control record All patients completed the 3-year follow-up, and there were no missing data.

Characteristic		IVS group	Control group	P-value	Test Statistic
Age at implant placement (years)		52.39 ± 11.47	55.85 ± 9.63	0.14	U=324 (Z=−1.48)
Preoperative PCR of remaining teeth (%)		26.58 ± 8.81^ *^	17.41 ± 10.27^ ***^	< 0.01^*^	U=629 (Z=3.28)
Preoperative BOP of remaining teeth (%)		21.26 ± 8.92^ **^	19.82 ± 12.89	0.56	U=456 (Z=0.58)
Preoperative alveolar crest bone width (mm)		9.35 ± 2.49	8.41 ± 1.71	0.11	U=677 (Z=1.60)
Sex, n (patient)	Male	8	9	0.57	OR = 0.70 [95% CI: 0.22–2.16]
	Female	23	18		
Diameter, n (implant)	Narrow (≤ 3.5 mm)	3	7	0.02^*^	OR = 0.27 [95% CI: 0.06–1.15]
	Regular (≥ 3.6 mm)	35	22		
Length, n (implant)	Short (≤ 8.0 mm)	5	7	0.33	OR = 0.48 [95% CI: 0.13–1.69]
	Medium (≥ 8.1 mm)	33	22		
Type of superstructure, n (implant)	Single	30	21	0.71	OR = 1.43 [95% CI: 0.32–6.36]
	Bridge	4	4		
Fixation method of prostheses, n (implant)	Cement	38	26	0.08	OR = 10.17 [95% CI: 0.50–205.14]
	Screw	0	3		

Postoperative outcomes

Implant survival at 3-years was 100% in both groups. All postoperative outcomes are described in Table 2. At 3-years, BOP was numerically lower in the IVS group, but the between-group difference did not reach statistical significance (p = 0.08). During surgery, all monitored parameters remained within predefined acceptable ranges, and no episodes met the instability criteria; no rescue airway maneuvers, vasoactive medications, or unplanned admissions were required.

**Table 2:** Postoperative outcomes Mann–Whitney U test was used for analysis. *P < 0.05. Data are expressed as mean (± standard deviation). A statistically significant difference was found between *, **, and ***. BOP: Bleeding on probing; IVS: Intravenous sedation; PCR: Plaque-control record All patients completed the 3-year follow-up, and there were no missing data.

Outcome		IVS group	Control group	P-value	Test Statistic
PCR of remaining teeth at 3 years post-superstructure placement (%)		14.94 ± 6.80^ *^	8.52 ± 7.18^ ***^	< 0.01^*^	U=621 (Z=3.16)
BOP at 3 years post-superstructure placement (%)		9.81 ± 10.40^ **^	19.82 ± 16.13	0.08	U=413 (Z=−1.75)
Alveolar crest bone width at 3 years post-superstructure placement (mm)		9.34 ± 2.18	7.95 ± 1.37	0.02^*^	U=735 (Z=2.33)
Apical bone width at 3 years post-superstructure placement (mm)		12.93 ± 2.37	13.08 ± 1.84	0.76	U=527 (Z=−0.31)
Horizontal bone loss at 3 years post-superstructure placement (mm)		0.04 ± 0.24	0.14 ± 0.43	0.01^*^	U=347 (Z=−2.58)
Maintenance visit within 6 months, n (patient)	Yes	30	17	< 0.01^*^	OR = 17.65 [95% CI: 2.08–150.00]
	No	1	10		
Peri-implant mucositis, n (implant)	Yes	12	7	0.78	OR = 1.32 [95% CI: 0.44–3.96]
	No	26	20		
Additional implant placement, n (patient)	Yes	17	3	< 0.01^*^	OR = 17.00 [95% CI: 3.81–75.89]
	No	7	21		

PROs

PRO scores are summarized in Table 3. The full author-developed questionnaire is provided in Appendix 1 (Table 4). Retrospectively reported preoperative anxiety (Q1) did not differ significantly between groups. The IVS group reported greater relaxation during treatment (Q2) and provided more favorable ratings for pain perception (Q3). Scores for awareness during the procedure (Q4) and time to leave the clinic after surgery (Q5) also differed significantly between groups. Additional subjective outcomes, including intraoperative discomfort (Q9), memory during treatment (Q10), and postoperative physical condition (Q11), also differed significantly between groups.

**Table 3:** Patient-reported outcome questionnaire (14 items) and mean scores of IVS vs. control groups at 3-year follow-up (single clinic, Japan) 4: Strongly positive/No symptoms, 3: Fairly positive/Slight symptoms, 2: Moderately positive/Some symptoms, 1: Slightly negative/Fair symptoms, 0: Strongly negative/Severe symptoms Mann–Whitney U test was used for analysis. *P < 0.05 Data are expressed as mean ± standard deviation. IVS, intravenous sedation All patients completed the 3-year follow-up, and there were no missing data.

Item	Scores of the IVS group	Scores of the Control group	P-value	Test Statistic
Q1. Level of preoperative anxiety	1.44 ± 0.91	2.05 ± 1.23	0.09	U=310 (Z=−1.70)
Q2. Relaxation during treatment	3.38 ± 0.78	1.65 ± 1.09	< 0.01^*^	U=755 (Z=5.24)
Q3. Perception of pain during treatment	3.94 ± 0.25	2.75 ± 0.99	< 0.01^*^	U=735 (Z=4.93)
Q4. Clarity of consciousness	0.30 ± 0.47	3.65 ± 0.61	< 0.01^*^	U=0 (Z=−6.52)
Q5. Recovery time after treatment	2.79 ± 0.79	3.65 ± 0.49	< 0.01^*^	U=148 (Z=−4.21)
Q6. Satisfaction after treatment	3.50 ± 0.61	3.55 ± 0.83	0.24	U=343 (Z=−1.17)
Q7. Willingness to use IVS again	3.18 ± 0.61	2.4 ± 0.88	< 0.01^*^	U=642 (Z=3.48)
Q8. Perceived effectiveness of implant treatment	3.65 ± 0.55	3.35 ± 0.49	0.06	U=539 (Z=1.88)
Q9. Discomfort during treatment	3.91 ± 0.39	3.25 ± 0.79	< 0.01^*^	U=647 (Z=3.56)
Q10. Memory during treatment	3.85 ± 0.37	1.15 ± 0.67	< 0.01^*^	U=837 (Z=6.52)
Q11. Physical condition after surgery	3.24 ± 0.77	2.15 ± 0.75	< 0.01^*^	U=707 (Z=4.50)
Q12. Short-term side effects	3.94 ± 0.25	3.55 ± 0.81	0.13	U=516 (Z=1.51)
Q13. Concentration after surgery	3.53 ± 0.67	3.35 ± 0.96	0.83	U=432 (Z=0.21)
Q14. Future use possibility	3.23 ± 0.61	2.5 ± 0.83	< 0.01^*^	U=637 (Z=3.40)

## Discussion

In this study, we compared implant therapy outcomes with and without IVS and found a 100% 3-year implant survival rate in both groups. A previous nationwide registry study [23] reported early implant loss in 4.4% patients; however, the favorable survival observed here may be attributable to the exclusion of patients with smoking habits, diabetes, or periodontal risk factors.

Before implant placement, the PCR of the remaining teeth was significantly higher in the IVS group than in the control group. This discrepancy may reflect heightened dental anxiety or resistance to prophylactic interventions among candidates for IVS, resulting in suboptimal oral hygiene at baseline. However, within-group comparisons revealed significant improvements over time in the IVS group: both PCR and BOP significantly decreased from baseline to 3-years post-treatment. In contrast, although the control group also exhibited a significant improvement in PCR, the change in BOP was not statistically significant. These findings suggest that peri-implant parameters at 3-years tended to favor the IVS group; however, the between-group difference in BOP at 3-years did not reach statistical significance. Therefore, given the observational study design, these results do not establish a causal effect of IVS on oral hygiene behavior or maintenance adherence.

The IVS group exhibited significantly poor baseline plaque control, which complicates the interpretation of longitudinal improvement; a part of this change may simply reflect regression to the mean rather than a true behavioral effect. Moreover, because this retrospective cohort lacked matching or multivariable adjustment, residual confounding by baseline hygiene status, implant diameter, or psychological profiles is likely. Together, these considerations support an associational rather than a mechanistic reading of the present findings.

Regular maintenance has been established as a crucial factor in the mid-term success of implant therapy. Greenstein et al. reported peri-implant mucositis and peri-implantitis in 46-63% and 19-23% patients, respectively, even among correctly placed implants, emphasizing the need for professional maintenance every 3-6 months [24]. Regular maintenance is widely advocated as critical for mid-term peri-implant health, and our cohort showed higher rates of attendance at maintenance visits in the IVS group. Dental implants are an excellent treatment modality, but are not superior to healthy natural teeth and are associated with biological and technical complications, underscoring the need for careful mid-term monitoring [25]. In the present study, the number of patients maintaining regular visits was significantly greater in the IVS group than in the control group. Patients who require IVS may represent a distinct subgroup that is often characterized by greater treatment complexity, higher cost, or heightened anxiety; therefore, they may be more likely to continue regular recall because of increased awareness of treatment investment or postoperative caution. Accordingly, IVS may serve as a proxy marker of patient characteristics rather than a causal driver of maintenance behavior.

Moreover, patients in the IVS group underwent significantly more additional implant placements, particularly those with two or more missing teeth. This observation is compatible with lower procedural anxiety among patients opting for IVS; however, selection and behavioral factors may also explain the higher acceptance of additional treatment, and causal attributions cannot be made. Walton et al. reported that concerns regarding surgical risk were the most common reason for declining implant therapy (43%) [26]. IVS may reduce such concerns, thus encouraging treatment acceptance. Supporting this notion, Morino et al. identified that patients receiving local anesthesia in combination with IVS showed significantly reduced sympathetic activity and lower salivary stress markers than did those receiving local anesthesia alone, indicating a quantifiable reduction in perioperative stress [27]. The current findings, including higher patient-reported scores for relaxation, reduced pain, intraoperative comfort, and willingness for future reuse, align with these observations.

While the diameter, length, and placement site of implants had no significant effect on survival outcomes in this study, patients undergoing IVS tended to receive implants with significantly larger diameters. As reported by Mously et al., wider implants are associated with higher survival rates [28]. It is conceivable that these favorable mechanical factors contributed to the observed reduction in bone loss in the IVS group.

This study has certain limitations. It was a small retrospective exploratory cohort study without randomization or multivariable adjustment, and residual confounding from baseline imbalances or unmeasured factors cannot be ruled out. Accordingly, the findings should be interpreted as associations rather than causal relationships. Second, the cohort was limited to medically healthy adults and excluded augmented sites, restricting generalizability. The strict eligibility criteria (non-smokers, no systemic disease, posterior implants placed in native bone without augmentation) likely selected a lower-risk cohort, which may yield higher survival and fewer complications than typical implant populations. In addition, requiring ≥3-year follow-up may select patients who are more engaged with care; therefore, the observed association between IVS and maintenance attendance may not generalize to less adherent or higher-risk populations. Third, the 3-year observation period was adequate for evaluating early stability but insufficient to capture long-term complications. In addition, multiple comparisons, use of a non-validated retrospective questionnaire, and the lack of formal inter-/intra-rater reliability assessment for radiographic measurements may increase imprecision, so small absolute differences (e.g., 0.10 mm) should be interpreted cautiously. Nevertheless, the complete follow-up and standardized protocol represent major strengths. Future studies with larger, more diverse populations and long-term designs incorporating multivariable analysis are warranted.

## Conclusions

Over a 3-year period in this single-clinic retrospective cohort, IVS use was associated with higher maintenance attendance within 6 months after superstructure attachment and more favorable patient-reported perioperative experience, while implant survival remained 100% in both groups. Differences in peri-implant clinical and radiographic parameters generally favored the IVS group; however, the between-group difference in BOP at 3-years did not reach statistical significance, and the absolute difference in horizontal bone loss was small. Given baseline group differences, potential confounding by indication, multiplicity of analyses, and measurement limitations, these findings should be interpreted as associative and hypothesis-generating. Prospective multicenter studies with longer (5-10-year) follow-up are warranted.

## Appendices

**Table 4:** Appendix 1. Author-developed postoperative questionnaire (14 items) Response scale (0–4): 4 = Strongly positive / No symptoms; 3 = Fairly positive / Slight symptoms; 2 = Moderately positive / Some symptoms; 1 = Slightly negative / Fair symptoms; 0 = Strongly negative / Severe symptoms. Abbreviations: IVS, intravenous sedation.

Item	Question (English)	Scale (0–4)
Q1. Level of preoperative anxiety	How anxious did you feel before undergoing implant treatment?	0/1/2/3/4
Q2. Relaxation during treatment	How relaxed did you feel during the implant surgery?	0/1/2/3/4
Q3. Perception of pain during treatment	How much pain did you feel during the treatment?	0/1/2/3/4
Q4. Clarity of consciousness	How aware did you feel during the implant procedure?	0/1/2/3/4
Q5. Recovery time after treatment	How long did it take you to leave the clinic after the surgery?	0/1/2/3/4
Q6. Satisfaction after treatment	How satisfied are you overall with the implant treatment?	0/1/2/3/4
Q7. Willingness to use IVS again	Would you choose intravenous sedation again in the future?	0/1/2/3/4
Q8. Perceived effectiveness of implant treatment	Do you feel the implant treatment was effective?	0/1/2/3/4
Q9. Discomfort during treatment	Did you feel any discomfort during the implant surgery?	0/1/2/3/4
Q10. Memory during treatment	How much do you remember about what happened during the treatment?	0/1/2/3/4
Q11. Physical condition after surgery	How was your physical condition after the surgery?	0/1/2/3/4
Q12. Short-term side effects	Did you experience any side effects shortly after surgery?	0/1/2/3/4
Q13. Concentration after surgery	Was your ability to concentrate affected in the hours after surgery?	0/1/2/3/4
Q14. Future use possibility	Would you choose implant treatment again in the future?	0/1/2/3/4
